# Effects of Postural Changes Using a Standing Desk on the Craniovertebral Angle, Muscle Fatigue, Work Performance, and Discomfort in Individuals with a Forward Head Posture

**DOI:** 10.3390/healthcare12232436

**Published:** 2024-12-04

**Authors:** Hyunju Lee, Yongwoo Lee

**Affiliations:** 1 Department of Physical Therapy, Graduate School, Sahmyook University, Seoul 01795, Republic of Korea; qkqh55088@syuin.ac.kr; 2 Department of Physical Therapy, College of Health and Welfare, Sahmyook University, Seoul 01795, Republic of Korea

**Keywords:** ergonomics, working environment, muscle fatigue, work performance, posture

## Abstract

Forward head posture is common among office workers who sit for long periods of time and is one of the main causes of neck pain. This study aimed to assess the effects of utilizing a standing desk on the craniovertebral angle, muscle fatigue, work performance, and discomfort in individuals with forward head posture. Twenty-four participants meeting the inclusion criteria were randomly assigned to either a traditional desk group (*n* = 12) or a standing desk group (*n* = 12). Each group engaged in 30 min of computer typing. To evaluate the impact of posture change using a standing desk, pre- and post-experiment discomfort surveys were conducted, and the craniovertebral angle and muscle fatigue were measured throughout the experiment. Work performance was assessed post-experiment based on the work completed by the participants. Intra-group analysis of the craniovertebral angle showed a significant decrease in the traditional desk group (*p* < 0.05) and a significant increase in the standing desk group (*p* < 0.05). When comparing the craniovertebral angle between the two groups, a significant difference was observed (*p* < 0.05). Additionally, significant differences in muscle fatigue, particularly in the levator scapulae muscles, were observed between the two groups (*p* < 0.05). The traditional desk group reported increased discomfort in the neck and shoulders (*p* < 0.05). These findings suggest that utilizing a standing desk can positively impact posture, reduce muscle fatigue, and alleviate discomfort in individuals with forward head posture, potentially serving as an intervention to prevent musculoskeletal disorders.

## 1. Introduction

Advances in technology have revitalized science, medicine, and economies, bringing significant benefits to individuals, businesses, and public and private enterprises. However, these developments have simultaneously brought about an increase in sedentary employment, inactive leisure activities, and sedentary behavioral patterns [1,2]. This leads to weight gain, decreased physical strength, and fatigue, and increases the risk of various diseases, such as cardiovascular disease, respiratory failure/insufficiency, type 2 diabetes, osteoporosis, dementia, and cancer [3,4,5]. Although prolonged sedentary lifestyles have been shown to increase the risk of disease and death, office workers spend 70–80% of their working hours sitting [6]. This increase places a continuous load on the spine, affecting anatomical structures, greatly increasing the risk of neck and shoulder pain, and increasing the incidence of forward head posture [7,8]. The most common method to determine the severity of forward head posture is measurement of the craniovertebral angle, with smaller values indicating poorer posture [9].

Sitting for long periods increases the risk of neck and shoulder pain [10], and incorrect posture related to this is forward head posture [7]. This posture is common among office workers [11], with the head pushed out in front of the center of the shoulders, shifting the center of gravity forward. To compensate for this, the trunk is moved backward, and the shoulders are rolled forward [7]. This causes muscle imbalance, pain, fatigue, and limitations in the joint range of motion [12]. Additionally, the activity of the posterior muscles of the neck increases rapidly, and high muscle fatigue is observed in the upper trapezius muscle [13], and the average length of muscle fibers contributing to the extensor torque of the atlantooccipital joint is reduced. This causes neck and shoulder pain [14]. Moreover, the pressure within the intervertebral disc increases twice as much as that in the standing position, increasing the load on the joints [15,16] and causing musculoskeletal discomfort, such as pain or discomfort in the neck, shoulders, and lower back [16]. Several studies have addressed these issues.

To prevent problems caused by sitting for long periods, it is recommended to take breaks or perform light stretching. According to a study by Ding, Cao [17], when working while sitting for a long time, standing up once every 40 min and stretching for 5 min can reduce fatigue of the upper trapezius and latissimus dorsi muscles. According to a study by Amoudi and Ayed [18], when muscle stretching is performed twice a day for five days a week, the joint range of motion is improved, and muscle fatigue is minimized. According to a study by Lee, Jeon [19], a modified cervical and shoulder traction exercise program restored neck alignment and reduced pain. However, these studies were conducted over a long period and did not monitor changes in participants’ postures in real-time. Additionally, studies on whether interventions can reduce musculoskeletal discomfort while maintaining work performance are lacking. Therefore, it is necessary to find a method that can be implemented in a real work environment with immediate effects.

Recently, various companies have introduced standing desks to prevent office workers from maintaining a sitting position for long periods [20]. Standing desks reduce sitting time by allowing users to alternate between sitting and standing positions [21], and are effective in reducing discomfort, physical fatigue, and sleepiness [22]. Additionally, various studies have shown that changes in posture that occur when moving from a sitting to a standing position using a standing desk improve the alignment of the entire spine, relieve pressure on the spine, and help prevent musculoskeletal diseases, such as neck and shoulder pain [22,23]. Because of these positive effects, standing desks are an important tool for preventing musculoskeletal disorders by preventing prolonged sitting postures. Various studies have been conducted among office workers.

Although changing posture using a standing desk rather than sitting for long periods has a positive effect on pain, discomfort, spinal alignment, and fatigue, there are some limitations. Most studies address ergonomic aspects and have used subjective indicators, such as questionnaires, rather than quantitative objective indicators, when evaluating pain, fatigue, or discomfort [24,25,26]. Additionally, although changing posture using a standing desk can have a significant impact on spinal alignment, including lower back, there are insufficient studies on forward head posture, which commonly occurs in individuals who sit for long periods.

This study aimed to explore the impact of standing desks, a widely adopted intervention for mitigating musculoskeletal issues among traditional desk and office workers, on key factors such as the craniovertebral angle (CVA)—a crucial indicator of head and neck alignment that signifies the severity of forward head posture (FHP). The study also examined how standing desks influence muscle fatigue, work performance, and discomfort, which are essential measures of physical well-being and productivity in individuals with prolonged sitting habits and FHP.

## 2. Materials and Methods

### 2.1. Participants

This study was conducted on healthy adult men and women in their 20s to 50s living in Seoul who met the inclusion criteria.

The inclusion criteria were as follows: ≥18 years of age, forward head posture with a CVA of ≤48°, and ability to use a computer. Those who were active in a sitting position for at least 30 h per week [27], did not have chronic musculoskeletal diseases, or had no experience using a standing desk were selected [28].

Exclusion criteria were patients with limitations in standing or sitting posture due to disease, those who had undergone neck or back surgery, and those with spinal disease (e.g., compression fractures and infection), neurological disorders, or cardiovascular disease within the past six months. Those who had pregnancy problems and were pregnant or preparing for pregnancy were selected [27,28,29].

The sample size of the study participants was determined using G*power 3.1 (G*power Ver 3.1.9.4, Germany, 2019). Through determination of CVA in a pilot experiment, an effect size of 0.88 was calculated, and 24 individuals were calculated using a statistical power of 0.75 and a significance level (α) of 0.05. Considering a dropout rate of 20%, 28 individuals was set as the number of participants. Before starting this study, the study was fully explained, and consent was obtained. Only those who signed the consent form participated in the experiment. The study was approved by the Sahmyook University Research Ethics Approval Committee (approval number: SYU 2023-07-014-001).

### 2.2. Procedure

This study used online promotional posts to recruit the participants. Promotional posts included ‘men/females > 18 years of age’, ‘individuals diagnosed with forward head posture or whose head is forward of the body’, ‘individuals who feel discomfort in their neck and shoulders when using a computer for long periods’, ‘individuals who sit for >30 h a week’, and ‘individuals with the potential to have a forward head posture’.

**Figure 1:**
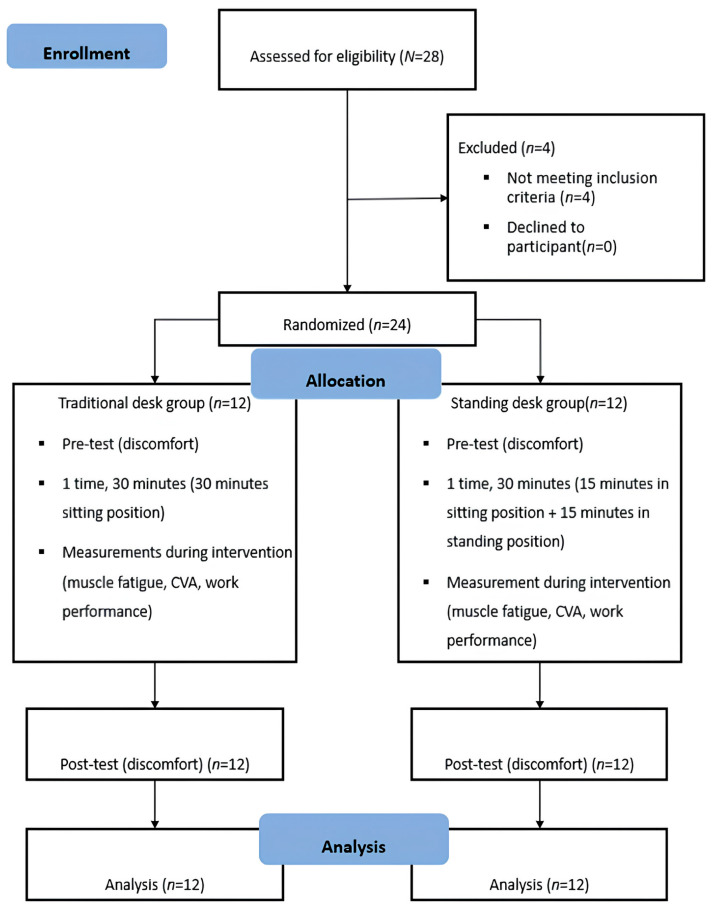
Flowchart.

We recruited 28 participants who expressed their intention to participate in this study, of whom 4 were eliminated because they did not meet the CVA inclusion criteria. All the experiments were conducted in a laboratory at the Sahmyook University. Before starting the experiment, general characteristics, such as sex, age, height, weight, and CVA, were recorded, and a questionnaire on discomfort before work was administered. The craniovertebral angle is the angle between the line connecting the C7 spinous process and the tragus area and a line horizontal to the ground [12,30,31], which was identified using the Cfix equipment (Cfix, W Surgitech Inc., Namyangju, Gyeonggido, Republic of Korea, 2022). The study participants were randomly assigned to the standing and traditional desk groups through separate drawing of lots for men and women and were assessed for CVA and muscle fatigue and work performance while working. Discomfort was measured using a post-work survey (Figure 1).

Before the experiment, the participants’ general characteristics, such as sex, age, height, weight, and CVA, were recorded, and the participants were randomly assigned to two groups by drawing lots separately for men and women. Subsequently, as a preliminary survey, the participants were asked to complete a questionnaire about their current discomfort.

The traditional desk group sat on a chair without a backrest or armrests and performed computer work for 30 min [32]. According to the ergonomics guide for computer-based work environments [33], the height of the computer monitor was set such that the top of the screen was parallel to eye level in a sitting position, and the chair was set such that the hip joints were bent at 100–120° and the knees were bent. It was set at a height below the buttocks [27]. Additionally, to accurately measure work performance, computer work was performed in a quiet environment without any disturbance.

The standing desk group performed typing work for 15 min in the same sitting position as the traditional desk group, and then typed work in a standing position for 15 min. In the standing position, the participant’s eye level was aligned with the top of the monitor, and when the elbow was bent at 90°, the desk was parallel to the forearm such that the desk was located underneath it [27,28]. To accurately measure work performance, computer work was performed in a quiet environment without any disturbance.

Immediately after completing 30 min of computer work, a post-survey was administered to the participants regarding their current discomfort.

### 2.3. CVA (Craniovertebral Angle)

In this experiment, CVA refers to the angle between the line connecting the C7 spinous process and tragus and the line parallel to the horizontal plane [11]. This suggests that the forward head posture worsens as the angle decreases. This was measured in real- time through Cfix (Cfix, W Surgitech Inc, Namyangju, Gyeonggido, Republic of Korea, 2022), which is a device that combines information and communication technology and a turtle-neck correction device. It measures the participant’s posture and movement information in real-time using an IMU sensor. To accurately measure movement, continuous acceleration values were measured at 60 Hz/s, and it was possible to classify normal individuals and turtle-neck patients with an accuracy of >85%. Additionally, the associated application allows real-time monitoring of posture and movement, and it provides an exercise program to improve turtle-neck. In this study, a plastic headband was worn, a ring was placed on the head, and the sensor was attached to the C7 spinous process. Before starting the typing task, a zero point was set by maintaining the posture of the head and back against the wall for 5 s, and the CVA was recorded in real-time at 10 Hz while performing the task.

**Figure 2:**
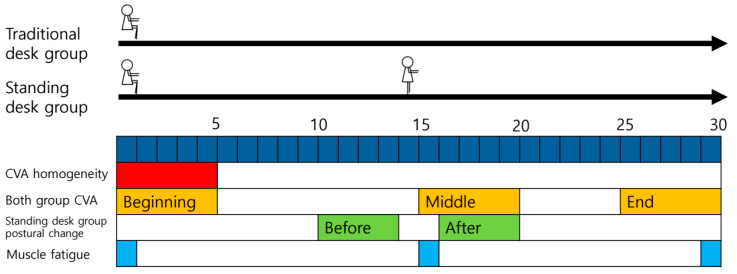
CVA and muscle fatigue visual representations. The arrows represent the timeline for each group.

The collected data were used to analyze four items. Homogeneity between groups was verified using the average value for the first 5 min, and the total change in CVA was calculated to compare the two groups. Additionally, the average values at 0–5 min, 15–20 min after the posture change occurred, and 25–30 min were calculated and referred to as the beginning, middle, and end, respectively. The CVAs of the two groups were compared over time. Finally, starting from 15 min when the standing desk group experienced a change in posture, the average values for 10–14 min and 16–20 min were calculated and compared before and after the change in posture occurred (Figure 2).

### 2.4. Muscle Fatigue

A surface electromyogram (Ultium Electromyogram; NORAXON, USA, 2021) was used to determine the fatigue level of the neck and shoulders of the participants with a forward head posture. The upper trapezius and levator scapulae muscles, which are related to neck and shoulder posture, were measured. The upper trapezius muscle is a muscle that shows high fatigue in participants with a forward head posture when working on a computer [34,35,36], and the levator scapulae muscle is a muscle that shows high fatigue in participants with a forward head posture. This muscle is stressed during long hours of computer work due to tension and can cause pain [36,37]. During the measurement, the distance between the centers of the ground electrodes was set to 2 cm. To reduce the skin’s resistance to an electromyography (EMG) signal, the EMG attachment area was removed by hair removal and exfoliation by wiping with an alcohol cotton pad [38,39]. The electrodes for each muscle were attached horizontally in the same direction as the muscle fibers at different locations [40,41].

Electromyography was performed throughout the experiment, and the collected data were analyzed using EMG software (MR 3.18 136 software; NORAXON, Scottsdale, AZ, USA, 2021). The sampling rate of the measured data was set to 2000 Hz, and the frequency bandpass was filtered to 10–500 Hz during signal processing to analyze the muscle fatigue [32,42].

Representative variables that can measure muscle fatigue in frequency analysis are the mean and median frequencies. Among them, the median frequency is a reliable indicator of muscle-related variables, such as muscle conduction velocity and temperature, and is known to be more resistant to noise than the mean frequency [43]. Homogeneity was verified using the average value of the median frequency.

As muscle fatigue accumulates, the EMG frequency moves to a lower frequency, and the mean and median frequencies decrease [44]. Therefore, the slope of the median frequency graph decreases and appears as a negative value. In this experiment, the slope value for the corresponding data was obtained from the values at 1, 15, and 29 min of 30 min of data (Figure 2) [32].

### 2.5. Work Performance

In this experiment, the work performance was measured by transferring text written in Word (MSO version 16.0. 2020; Microsoft Word 365) to another file. For right-handers, the text to be written was placed on the left half of the screen and an empty file was placed on the right. For left-handers, the text to be written was placed on the right half of the screen and an empty file was placed on the left. After aligning the screen with the dominant hand, the text was transcribed into a blank file [28]. The contents of the text were news articles in various fields, such as the economy, society, life/culture, and information technology/science, and were collected into one file.

Computer work performance was analyzed by dividing the number of typing and errors(typos) rate by the number of typing [28]. The number of typing was measured as the number of characters, excluding spaces in the text written throughout the work, and the error rate was the same as that in Formula (1).

(1) $$\mathrm{Error}\;\mathrm{rate}\;(\%)=\frac{number\;of\;typos}{number\;of\;typing}\times 100$$

### 2.6. Discomfort

A Standardized Nordic musculoskeletal questionnaire was used to measure physical discomfort before and after using traditional and standing desks. This questionnaire allows for easy comparison of studies on musculoskeletal symptoms. It is divided into two parts: a general questionnaire and a separate questionnaire on the back, neck, and shoulders [45,46]. The validity of this questionnaire was 87.2%, and its internal consistency reliability (Cronbach’s alpha) was 0.965–0.966 [47].

In this study, a general questionnaire translated into the Korean language from the standardized Nordic-style questionnaire described by Choi, Sung [48] and a questionnaire with additional items to measure the intensity of symptoms were used. The intensity of symptoms was expressed on a visual analog scale between 1–10 points. In this study, discomfort in the neck, shoulder, elbow, and wrist was measured. The sensitivity of this questionnaire was 73.9%, the specificity was 68.0%, the positive predictive value was 72.6%, and the negative predictive value was 69.5%.

### 2.7. Data Analysis

This study was analyzed using SPSS (IBM SPSS statistics version 22.0; IBM Co., Armonk, NY, USA, 2014). All data were tested for normality using the Shapiro–Wilk test and confirmed to be normally distributed. To confirm homogeneity between groups, the chi-squared test and an independent samples *t*-test were performed. An independent samples *t*-test was used to compare muscle fatigue, work performance and discomfort between the two groups, and a paired samples *t*-test was conducted to compare muscle fatigue and discomfort over time within the group. A mixed-design analysis of variance (ANOVA) was conducted to compare the differences in CVA between the two groups over three time points. The between-subject factor was group, and the within-subject factor was time. This analysis allowed for the examination of main effects of group and time, as well as their interaction effect (Group × Time). The assumptions of normality and sphericity were assessed prior to the analysis. For violations of sphericity, the Greenhouse–Geisser correction was applied. Post hoc pairwise comparisons were performed with Bonferroni adjustment to identify specific differences between groups and time points. The statistical significance level for all data was set at *p* < 0.05.

## 3. Results

### 3.1. General Characteristics of Research Subjects

The study subjects were 24 men and women: 12 in the standing desk group (4 men, 8 women) and 12 in the traditional desk group (4 men, 8 women). There was no significant difference between the standing desk group and the traditional desk group in all ranges, so the two groups were homogeneous (Table 1).

### 3.2. Measurement of Homogeneity for Each Measurement Item of Research Subjects

The first 5 min CVA, initial median frequency for assessing muscle fatigue, and discomfort of the two groups are shown in the following graph, and there was no significant difference (*p* > 0.05), so the two groups were homogeneous (Table 2).

### 3.3. Comparison of Total Variance in Craniovertebral Angle According to Intervention Method

**Figure 3:**
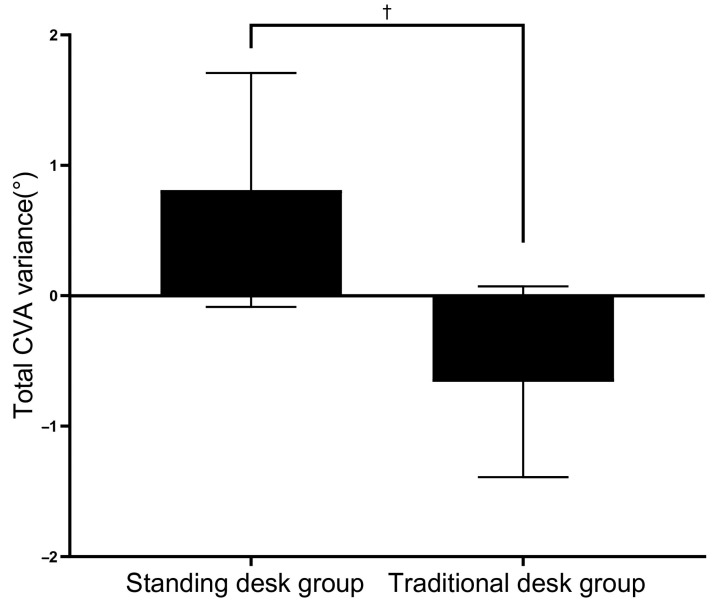
Total CVA variance. † (*p* < 0.05) Comparison of changes between groups.

In comparing the total variance in CVA according to the intervention method, there was a significant difference between the two groups: 0.81° for the standing desk group and –0.66° for the traditional desk group (*p* < 0.05) (Table 3) (Figure 3).

### 3.4. Comparison of Craniovertebral Angle According to Intervention Method

A mixed-design ANOVA demonstrated a significant interaction effect between group and time for CVA ($\eta^{2}$p = 0.47, *p* < 0.01), indicating differential changes in the craniovertebral angle over time between the standing desk and traditional desk groups. Specifically, the standing desk group exhibited a progressive increase in CVA from the beginning (45.58°) to the end (46.63°), while the traditional desk group showed a decrease from 45.98 ± 1.51° to 45.00 ± 2.20°. 

Post hoc analyses revealed no significant differences between the two groups at the beginning (*p* = 0.56) or middle (*p* = 0.37) time points. However, a significant difference was observed at the end of the study, with the standing desk group displaying a greater CVA compared to the traditional desk group (*p* = 0.04).

**Figure 4:**
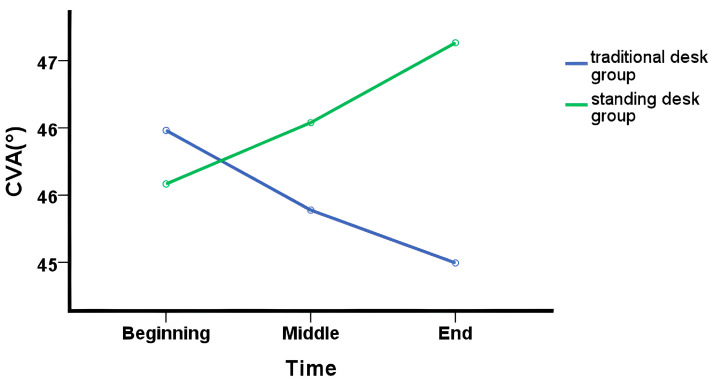
Changes in craniovertebral angle over time by group. CVA = craniovertebral angle.

No significant main effects were found for group ($\eta^{2}$p = 0.04, *p* = 0.37) or time ($\eta^{2}$p = 0.01, *p* = 0.73). (Table 4) (Figure 4).

The change in craniovertebral angle according to posture in the standing desk group significantly increased from 45.31° before standing up to 46.77° after standing up (*p* < 0.05) (Table 5).

### 3.5. Comparison of Muscle Fatigue Slope According to Intervention Method

When comparing the muscle fatigue slopes of the two groups, the standing desk group showed a significantly greater improvement in the right levator scapulae (0.53% vs. −3.83%, *p* < 0.05) and left levator scapulae (0.29% vs. −2.59%, *p* < 0.05) compared to the traditional desk group. However, there were no significant differences between the groups in the right upper trapezius (0.63% vs. −0.66%) or left upper trapezius (0.48% vs. −1.24%). These results suggest that the standing desk group experienced more favorable changes in the levator scapulae muscles (Table 6).

### 3.6. Comparison of Work Performance According to Intervention Methods

There was no significant difference in the number of typing in the work performance, with the standing desk group having 2868.42 strokes and the traditional desk group having 3085.08 strokes. The error rate was 0.33% for the standing desk group and 0.40% for the traditional desk group, with no significant difference (Table 7).

### 3.7. Comparison of Discomfort According to Intervention Method

In terms of neck discomfort, the standing desk group decreased from 4.00 to 3.50 points, while the traditional desk group increased significantly from 4.17 to 5.33 points (*p* < 0.05), with a significant difference between the two groups after work (*p* < 0.05).

For shoulder discomfort, the standing desk group increased from 3.92 to 4.25 points, whereas the traditional desk group showed a larger increase from 4.33 to 5.92 points (*p* < 0.05), with a significant difference between the two groups after work (*p* < 0.05).

There was no significant difference between the two groups for elbow and wrist discomfort (Table 8).

## 4. Discussion

This study sought to determine whether changing posture through a standing desk is an intervention that could prevent problems that occur when office workers with forward head posture work on computers for long time. Therefore, we investigated the CVA, muscle fatigue, work performance and discomfort of subjects with forward head posture when a group using a standing desk and a group using a traditional desk worked on a computer for 30 min. As a result, significant differences were seen in CVA, muscle fatigue of both levator scapulae muscles, and neck and shoulder discomfort.

The result of measuring CVA throughout the experiment in this study indicated that the standing desk group significantly increased due to posture changes, and the traditional desk group significantly decreased. There were also significant differences between the two groups.

Forward head posture commonly occurs in people who work on a computer while sitting for long periods of time, and its severity significantly increases with the time spent on the computer [49,50]. Additionally, when trunk flexion and neck flexion increase in a sitting position, the forward movement of the head increases [51], and even when there is no trunk flexion, when the arms are extended forward, neck flexion increases [15]. Additionally, forward head posture worsens when working on a computer while staring at a monitor [12]. In this study, the subjects performed computer work for 30 min while sitting with their arms out in front of the trunk and continuously staring at the monitor. Therefore, like previous studies, the traditional desk group in this study suffered from a worsening of forward head posture over time.

In a previous study, the CVA of subjects with forward head posture when sitting was 40.7° and when standing, the CVA was significantly different at 43.4°, and it was stated that neck alignment is affected by the posture of the trunk, pelvis, arms and legs [52]. The lumbopelvic sagittal alignment is different when sitting and standing, and the sagittal alignment of the cervical spine is influenced by the lumbopelvic sagittal alignment. Therefore, when sitting, the posterior tilt of the pelvis, a decrease in lumbar lordosis, and an increase in thoracic kyphosis occur, resulting in an increase in cervical lordosis. Conversely, when standing, the anterior tilt of the pelvis and lumbar lordosis increases, and thoracic kyphosis decreases, resulting in a decrease in cervical lordosis [53,54]. Therefore, when standing, the head moves backward compared to when sitting, that is, the degree of forward head posture decreases.

Through a study by Karakolis, Barrett [55], it was confirmed that lumbar flexion was significantly greater in a sitting position than in a standing position when working on a computer. Additionally, when sitting on a chair without a backrest, the kyphosis of the thoracic increases and the forward movement of the head increases [56]. Therefore, because the traditional desk group in this study performed work in a sitting position on a chair without a backrest, there was a decrease in lumbar lordosis, an increase in thoracic kyphosis and cervical lordosis, and as a result, the forward head posture worsened. On the other hand, in the standing desk group, there was an increase in lumbar lordosis and a decrease in cervical lordosis in the standing position, which is believed to have improved the forward head posture.

In a study by Choi and Hwang [57], it was confirmed that when subjects with forward head posture performed neck and chest stretching and muscle strengthening exercises four times a week for 10 weeks, CVA significantly increased from 59.19° to 63.97°. In a study by Abdollahzade, Shadmehr [58], it was confirmed that when posture correction exercises were performed on subjects with forward head posture three times a day for four weeks, CVA significantly increased from 46.21° to 51.12°. In a study by Kang and Yang [59], it was confirmed that when subjects with forward head posture were subjected to a neck and shoulder exercise program 2 to 3 times a week for 4 weeks, CVA significantly increased from 62.08° to 63.40°. Just as a long-term exercise program has a significant effect on improving the posture of subjects with forward head posture, we also found that changing posture through a standing desk even for a short period of time can have an effect on increasing CVA and improving immediate posture in subjects with forward head posture.

In this study, as a result of comparing the muscle fatigue of both levator scapulae and upper trapezius muscles in subjects with forward head posture, both levator scapulae muscles were measured to be significantly smaller.

Forward head posture causes excessive extension of the upper cervical and atlantooccipital joints. The muscles behind the cervical spine, such as the suboccipital, pectoralis major, upper trapezius, levator scapulae, and latissimus dorsi, are shortened, and the deep neck flexors, rhomboids, middle and lower trapezius, and serratus anterior muscles are weakened [59]. Janda noted that forward head posture is associated with increased tension, especially in the trapezius and levator scapulae muscles [37].

In this study, when computer work was performed for 30 min in each work environment, there was a change in muscle fatigue in subjects with forward head posture, especially significant in both levator scapulae muscles. This can be divided into changes in arm posture depending on posture and changes in CVA over time.

According to previous research, when performing computer work in a standing position, shoulder abduction of 5.6°, flexion of 4.9°, and lateral rotation of 7.2° occur, and in a sitting position, shoulder abduction of 10.5°, flexion of 18.8°, and lateral rotation of 17.2° occur [60]. Among these movements, the agonist muscle for lateral rotation of the scapula is the upper trapezius, and the antagonist muscle is the levator scapula. In subjects with forward head posture, muscle activity of the upper trapezius increases and shortening and tension of the levator scapulae muscle increase, causing limitations in lateral rotation [37,61]. Therefore, in order for subjects with forward head posture to perform computer work in a sitting position, lateral rotation of the shoulder increased, which increased the muscle activity of the upper trapezius muscle and placed a large load on the shortened and tense levator scapulae muscle, increasing muscle fatigue. In addition, it is thought that in the standing position, the lateral rotation of the scapula is reduced, the muscle activity of the upper trapezius muscle is reduced, and the load on the levator scapulae muscle is reduced, thereby reducing muscle fatigue.

In a previous study, the CVA of subjects with forward head posture was compared when sitting and standing, and the CVA of the sitting posture was 40.7° and the CVA of the standing posture was 43.4°, which showed a significant difference between the CVA when sitting and standing [9]. Additionally, when sitting and continuing to work on a computer, CVA significantly increases over time [50]. In another previous study, muscle activity of the upper trapezius muscle according to head position was measured in sitting and standing positions, and the muscle activity of the upper trapezius muscle increased as the head moved forward in both postures [62]. This increase in muscle activity can cause muscle fatigue [63], and muscle fatigue especially increases when performing repetitive tasks in a fixed posture [64]. Therefore, in this study, the traditional desk group maintained a sitting posture for a long time without much movement, and it is thought that muscle fatigue increased because the forward head posture worsened over time. On the other hand, in the standing desk group, posture changes occurred when standing up from sitting, and CVA decreased accordingly, which is believed to have reduced muscle fatigue.

In this study, the work performance of using a traditional desk and a standing desk was compared through the number of typing and the error rate, and there was no significant difference between the two groups.

Previous studies have shown that when using a standing desk, cognitive ability decreases in a standing position compared to a sitting position. This is because the degree of freedom (DOF) increases while standing, increasing the cognitive burden to maintain posture [65]. However, in a study by Karakolis and Callaghan [25], there was no significant change in work performance, and this was stated to be because the study was conducted in a laboratory. A study by [66] stated that there are differences in typing speed and errors depending on the type of standing desk, but this may be due to unfamiliarity with new work equipment. Therefore, this study was conducted in a laboratory for a short period of time, and both groups consisted of adults with no cognitive function problems and were proficient in using computers, so it is believed that there was no significant difference in work performance despite changes in posture. Also, because the initial performance was not measured, the typing skills of the two groups may not equal, which may have affected the experimental results.

In this study, as a result of comparing neck, shoulder, elbow, and wrist discomfort in the standing desk group and the traditional desk group, the traditional desk group had significantly increased neck and shoulder discomfort, and the neck and shoulder discomfort was significantly higher than in the standing desk group.

Incorrect posture, such as forward head posture, causes excessive tension in surrounding muscles, which induces abnormal force generation in the musculoskeletal system and causes pain. In particular, when working on a computer for a long time, excessive tension occurs in the upper trapezius muscle, which leads to pain and discomfort [28]. Additionally, when performing computer work for a long period of time in a sitting position and moving the head forward, the muscles of the cervical spine receive a large load to support the posture [67]. In particular, in subjects with a forward head posture, as the forward movement of the head increases due to the extension of the upper cervical vertebrae and the flexion of the lower cervical vertebrae, the tension in the neck extensor muscles increases and the compressive force on the cervical joint surface increases [68].

When using a traditional desk, the forward movement of the head increases over time, causing the center of gravity to move forward, and the activation and tension of the muscles behind the head and neck increase to compensate [50]. As muscle contraction continues in a fixed state to maintain the forward head posture, blood circulation decreases and waste products accumulate, irritating the nerves. This causes blood vessels to contract abnormally, further worsening blood circulation problems, and pain and discomfort increase over time [69]. In this study, it is believed that the forward head posture of the group that used traditional desk worsened over time, and that muscle contraction in the neck and shoulders increased to maintain this, resulting in increased physical discomfort.

In a previous study, it was suggested that in order to reduce discomfort and pain when working on a computer for a long time, you should maintain an appropriate posture and exercise or stretch occasionally to avoid maintaining one posture for a long time [50]. In this study, a standing desk that allows alternating between sitting and standing positions was selected as an intervention to reduce the time spent in a sitting position. Standing desks significantly reduce the sitting time of office workers during work hours [70] and improve spinal alignment through changes in posture [22,71]. Kyphosis of the lumbar spine and forward movement of the head are less in the standing position than in the sitting position, which means that working on a computer in the standing position requires less physical load than in the sitting position [65]. In addition, according to previous studies, muscle activity in the back of the neck and trunk decreases when standing compared to sitting, and performing work in a standing position rather than sitting is beneficial in reducing body load [72].

In a study by Nevala and Choi [73], working on a computer at a standing desk for 42 min resulted in less discomfort than at a traditional desk, especially in the upper extremities. In a study by EF Graves, C Murphy [74], discomfort in the neck, shoulders, and upper back decreased when office workers used a standing desk for 8 weeks. In a study by Ma, Ma [75], pain in the neck and shoulders was significantly reduced when a standing desk was used for 3 months. Similar to the results of these previous studies, this study indicated that the change in posture through the standing desk improved the overall alignment of the spine and reduced forward head posture, thereby reducing the load on the neck and shoulders, resulting in less discomfort than in the traditional desk group.

Through this study, it was confirmed that changing posture through a standing desk was effective in increasing CVA, reducing muscle fatigue, and reducing neck and shoulder discomfort in subjects with forward head posture. Working on a computer in the same position for a long time can cause musculoskeletal disorders, including forward head posture. Therefore, we suggest that periodically changing posture through a standing desk is effective in preventing problems that occur when office workers with forward head posture work on computers for long periods of time, and in maintaining their health and correct posture. In addition, we further propose a standing desk as an intervention to prevent musculoskeletal disorders, such as forward head posture and fatigue caused by long-term computer use. Therefore, it is believed that follow-up research is needed to determine the effect of workers with forward head posture using standing desks in actual work environments and whether the incidence of forward head posture can be reduced when actual workers use standing desks for long periods of time.

This study has some limitations. First, in this study, all subjects performed a typing task for 30 min. It is believed that it is a short period of time for muscle fatigue, discomfort, and work performance to change significantly. Therefore, it is difficult to say that the same results will be obtained after using a standing desk for a long time. Second, the standing desk group in this experiment sat for 15 min and then worked while standing for 15 min. Since the posture is changed only once, there is a possibility that different results would be obtained if posture is changed repeatedly over a long period of time. Third, it is difficult to generalize the research results because the number of subjects was insufficient. In addition, subjects with forward head posture were selected, but the degree varied. Fourth, because equipment was attached to measure CVA and muscle fatigue during the experiment, the subjects may have consciously corrected their posture and changed their muscle tension. Fifth, standing for a long time may cause discomfort in the lower extremity and back, but this was not measured in this experiment. Lastly, in this experiment, a chair without a backrest and armrests was used, but in actual work environments, chairs with a backrest and armrest are usually used, which affects the alignment posture, including the lower back and neck. Therefore, in follow-up studies, the measurement time should be increased, posture changes should be made several times, and the experiment should be conducted under conditions similar to those in the actual work environment.

## 5. Conclusions

This study sought to determine the effect of changes in posture through a standing desk on CVA, muscle fatigue, work performance and discomfort in subjects with forward head posture. The results are as follows.

In the CVA comparison, the traditional desk group’s CVA significantly decreased, and the standing desk group’s CVA significantly increased. Also, when comparing the CVA of the two groups, there was a significant difference. When comparing muscle fatigue between the groups, there was a significant difference in both levator scapulae muscles. There was no significant difference when comparing work performances between the two groups. Discomfort in the traditional desk group significantly increased in the neck and shoulders, and when comparing the two groups, there was a significant difference in the neck and shoulders.

This result shows that using a standing desk can improve cervical alignment, reduce muscle fatigue, and decrease neck and shoulder discomfort in office workers with forward head posture. While these benefits were observed in the short term, further research with longer durations and larger sample sizes is needed to confirm the long-term effects and applicability in real-world settings.

Through this investigation, this study proposes a standing desk as a work environment for patients with forward head posture and is expected to be of basic help in research to determine the effectiveness of standing desks as an intervention to prevent musculoskeletal diseases including forward head posture.

## Figures and Tables

**Table 1:** General characteristics.

	Standing Desk Group (*n* = 12)	Traditional Desk Group (*n* = 12)	*χ*^2^/*t*(*p*)
Sex (Male/Female)	4/8	4/8	0.000 ^b^ (1.000)
Age (year)	26.67 ± 1.72 ^a^	26.67 ± 2.31	−0.213 ^c^ (0.831)
Height (cm)	163.92 ± 7.89	163.83 ± 8.88	−0.058 (0.954)
Weight (kg)	59.25 ± 11.63	60.17 ± 12.69	−0.145 (0.885)
BMI (kg/m^2^)	21.09 ± 2.86	22.16 ± 2.52	−0.144 (0.885)
CVA (°)	45.73 ± 1.61	45.97 ± 1.44	−0.058 (0.977)

CVA = craniovertebral angle; BMI = body mass index; ^a^ Mean ± standard deviation; ^b^ Chi-squared test; ^c^ Independent-sample *t*-test.

**Table 2:** Measurement of homogeneity for each measurement item of research subjects.

		Standing Desk Group (*n* = 12)	Traditional Desk Group (*n* = 12)	*t*(*p*) ^b^
First 5 Minutes CVA (°)		45.58 ± 1.80 ^a^	45.98 ± 1.51	−0.231 ^b^ (0.843)
Initial median frequencies (Hz)	Rt. LS	63.43 ± 12.59	66.13 ± 9.17	−0.635 (0.551)
	Rt. UT	68.05 ± 8.48	65.70 ± 6.64	−0.635 (0.551)
	Lt. LS	55.42 ± 12.15	58.86 ± 8.96	−0.924 (0.378)
	Lt. UT	63.53 ± 8.36	60.72 ± 8.22	−0.404 (0.713)
Discomfort (point)	Neck	4.00 ± 1.47	4.17 ± 2.29	−0.059 (0.953)
	Shoulder	3.92 ± 2.11	4.33 ± 1.72	−0.799 (0.443)
	Elbow	1.00 ± 0.95	1.24 ± 1.05	−0.422 (0.719)
	Wrist	3.00 ± 2.59	2.75 ± 2.34	−0.147 (0.887)

CVA = craniovertebral angle; Rt = right; Lt = left; LS = levator scapulae; UT = upper trapezius. ^a^ Mean ± standard deviation; ^b^ Independent-sample t-test.

**Table 3:** Comparison of total variance in craniovertebral angle according to intervention method.

	Standing Desk Group (*n* = 12)	Traditional Desk Group (*n* = 12)	*t*(*p*) ^b^
Total CVA variance	0.81 ± 0.90 ^a^	−0.66 ± 0.73	−4.403 (0.00)

CVA = craniovertebral angle. ^a^ Mean ± standard deviation. ^b^ Significance probability after between-group experiment.

**Table 4:** Comparison of craniovertebral angle (CVA) between groups over time using mixed ANOVA.

	Group	Time			Mixed ANOVA (*p*-Value)		
		Beginning	Middle	End	$\mathrm{Interaction}\;(\mathrm{Group}\;\mathrm{and}\;\mathrm{Time})\;\mathrm{Effect}\;\eta^{2}$p (*p* Value)	$\mathrm{Group}\;(G)\;\mathrm{Effect}\;\eta^{2}$p (*p* Value)	$\mathrm{Time}\;(T)\;\mathrm{Effect}\;\eta^{2}$p (*p* Value)
CVA (°)	Standing desk group	45.58 ± 1.80 ^a^	46.04 ± 1.52	46.63 ± 1.40	0.47 (0.00) ^†^	0.04 (0.37)	0.01 (0.73)
	Traditional desk group	45.98 ± 1.51	45.38 ± 1.98	45.00 ± 2.20			

CVA = craniovertebral angle. ^a^ Mean ± standard deviation. ^†^ *p* < 0.05.

**Table 5:** The change in craniovertebral angle according to posture in the standing desk.

	Before Standing Up	After Standing Up	*t*(*p*) ^b^
CVA (°) according to position	45.31 ± 1.73 ^a^	46.77 ± 1.52	−7.004 (0.00) ^†^

CVA = craniovertebral angle. ^a^ Mean ± standard deviation. ^b^ Significance probability after between-group experiment. ^†^ (*p* < 0.05) Between-group comparison.

**Table 6:** Comparison of muscle fatigue slope according to intervention method.

Muscle Fatigue Slope (%)	Standing Desk Group (*n* = 12)	Traditional Desk Group (*n* = 12)	*t*(*p*) ^b^
Rt. LS	0.53 ± 3.07 ^a^	−3.83 ± 2.97	−3.529 (0.002) ^†^
Rt. UT	0.63 ± 3.21	−0.66 ± 2.54	−1.087 (0.289)
Lt. LS	0.29 ± 2.56	−2.59 ± 2.52	−2.774 (0.011) ^†^
Lt. UT	0.48 ± 3.06	−1.24 ± 1.60	−1.725 (0.103)

Rt = right; Lt = left; LS = levator scapulae; UT = upper trapezius; ^a^ Mean ± standard deviation. ^b^ Significance probability after between-group experiment. ^†^ (*p* < 0.05) Between-group comparison.

**Table 7:** Comparison of work performance according to intervention methods.

	Standing Desk Group (*n* = 12)	Traditional Desk Group (*n* = 12)	*t*(*p*) ^b^
Number of typing (strokes)	2868.42 ± 751.00 ^a^	3085.08 ± 643.96	0.759 (0.456)
Error rate (%)	0.33 ± 0.19	0.40 ± 0.18	0.831 (0.415)

^a^ Mean ± standard deviation. ^b^ Significance probability after between-group experiment.

**Table 8:** Comparison of discomfort according to intervention method.

NRS (Points)		Standing Desk Group (*n* = 12)	Traditional Desk Group (*n* = 12)	*t*(*p*) ^b^
Neck	Before	4.00 ± 1.48 ^a^	4.17 ± 2.29	−0.059 (0.953)
	After	3.50 ± 1.62	5.33 ± 2.35	−2.224 (0.037) ^†^
	Difference before and after	0.50 ± 1.16	−1.17 ± 1.53	
	*t*(*p*)	1.483 (0.166)	−2.646 (0.023) *	
Shoulder	Before	3.92 ± 2.11	4.33 ± 1.72	−0.799 (0.443)
	After	4.25 ± 1.60	5.92 ± 2.34	2.100 (0.047) ^†^
	Difference before and after	−0.33 ± 1.72	−1.58 ± 1.37	
	*t*(*p*)	−0.670 (0.517)	−3.978 (0.002) *	
Elbow	Before	1.00 ± 0.95	1.24 ± 1.08	−0.422 (0.719)
	After	1.25 ± 1.29	1.25 ± 1.14	0.000 (1.000)
	Difference before and after	−0.50 ± 1.00	−0.67 ± 0.44	
	*t*(*p*)	−1.000 (0.339)	−0.052 (0.959)	
Wrist	Before	3.00 ± 2.59	2.75 ± 2.34	−0.147 (0.887)
	After	3.25 ± 2.05	3.25 ± 2.34	0.000 (1.000)
	Difference before and after	0.25 ± 1.48	−0.50 ± 1.67	
	*t*(*p*)	−0.583 (0.571)	−1.032 (0.324)	

NRS: Numeric rating scale; ^a^ Mean ± standard deviation. ^b^ Significance probability after between-group experiment. * (*p* < 0.05) Before and after comparison within the group. ^†^ (*p* < 0.05) Between-group comparison.

## Data Availability

The data that support the findings of this study are available from the corresponding author [A.M.] upon reasonable request.
